# PART1 expression is associated with poor prognosis and tumor recurrence in stage I-III non-small cell lung cancer: Retraction

**DOI:** 10.7150/jca.85747

**Published:** 2023-05-15

**Authors:** Ming Li, Weiwei Zhang, Shenjun Zhang, Changhui Wang, Yinping Lin

**Affiliations:** 1 Department of Respiratory Medicine, Shanghai 10th People's Hospital, Tongji University, Shanghai 200072, China.; 2 Department of Endocrinology, Xinhua Hospital, Shanghai Jiaotong University School of Medicine, Shanghai 200092, China; 3 Institute of Biochemistry and Biology, Shanghai Institutes for Biological Sciences, Chinese Academy of Sciences, Shanghai 200031, China.

After the article was published, the corresponding author found that the conclusions of the article were incomplete. To ensure scientific rigor, the corresponding author decided to retract the article. The first author, Dr. Li, agreed to retract the article, and the other authors did not reply whether they agreed to retract the article.

For the sake of scientific rigor, the corresponding author of the article, made a final decision to retract this paper.

